# Therapeutic implications of galectin-3 in patients with atrial fibrillation

**DOI:** 10.1038/s41598-022-04894-9

**Published:** 2022-01-17

**Authors:** Kwang-No Lee, Do Young Kim, Ki Yung Boo, Yun Gi Kim, Seung-Young Roh, Yong-Soo Baek, Dong-Hyeok Kim, Dae In Lee, Jaemin Shim, Jong-Il Choi, Gyo-Seung Hwang, Young-Hoon Kim

**Affiliations:** 1 Department of Cardiology, Ajou University School of Medicine, World cup-ro 164, Yeongtong-gu, Suwon-si, Gyeonggi-do 16499 Republic of Korea; 2 Division of Cardiology, Department of Internal Medicine, Hallym University Dongtan Sacred Heart Hospital, Keunjaebong-gil 7, Hwaseong-si, Gyeonggi-do 18450 Republic of Korea; 3 Division of Cardiology, Department of Internal Medicine, Jeju National University Hospital, Aran 13-gil 15, Jeju-si, Jeju-do 63241 Republic of Korea; 4 Division of Cardiology, Department of Internal Medicine, Korea University Anam Hospital, Goryeodae-ro 73, Seongbuk-gu, Seoul, 02841 Republic of Korea; 5 Division of Cardiology, Department of Internal Medicine, Korea University Guro Hospital, Gurodong-ro 148, Guro-gu, Seoul, 08308 Republic of Korea; 6 Division of Cardiology, Department of Internal Medicine, Inha University Hospital, Inhang-ro 27, Jung-gu, Incheon, 22332 Republic of Korea; 7 Division of Cardiology, Department of Internal Medicine, Ewha Womans University Medical Center, Gonghang-daero 260, Balsan 1(il)-dong, Gangseo-gu, Seoul, 07804 Republic of Korea; 8 Division of Cardiology, Department of Internal Medicine, Chungbuk National University Hospital, 1sunhwan-ro 776, Seowon-gu, Cheongju-si, Chungcheongbuk-do 28644 Republic of Korea

**Keywords:** Cardiology, Interventional cardiology

## Abstract

Atrial fibrosis can present as an arrhythmogenic substrate that is correlated with higher recurrence after catheter ablation for atrial fibrillation. Galectin-3, a beta-galactoside-binding lectin, is highly expressed and secreted from macrophages and is important in inflammation and fibrosis. We assessed the clinical implications of serum galectin-3 in patients with atrial fibrillation. This was a prospective cohort study of consecutive patients who underwent radiofrequency catheter ablation in a tertiary referral center from February 2017 to September 2017. Intracardiac blood sampling, echocardiographic measurements, magnetic resonance imaging with late gadolinium enhancement, electrophysiologic testing, and endocardial voltage mapping were consistently implemented in 75 patients before the ablation. Serum galectin-3 level was higher in patients with diabetes mellitus and was correlated with values that indicated the left atrial size. During a median 14 months of follow-up, atrial tachyarrhythmia recurred in 27% of patients. In multivariable Cox regression analysis, non-paroxysmal atrial fibrillation (hazard ratio 6.8; 95% confidence interval 1.6–28.9) and higher galectin-3 levels (hazard ratio 1.3; 95% confidence interval 1.0–1.7) were associated with increased risk of recurrence. Serum galectin-3 may be a prognostic biomarker for risk stratification in patients with atrial fibrillation planned catheter ablation.

## Introduction

Atrial fibrillation is the most common type of sustained cardiac arrhythmia, and the prevalence increases with corresponding increases in age^1^. Atrial fibrillation not only increases the risk of morbidity and mortality, but also decrease the quality of life^2^. Since the recognition of triggers that initiate atrial fibrillation from the pulmonary veins, electrical isolation of the pulmonary veins using radiofrequency catheter ablation has become an important therapeutic option in patients with recurrent, drug-refractory symptoms^2,3^.

However, the efficacy of catheter ablation is difficult to predict in individual patients. Atrial structural remodeling with fibrosis contributes to the pathophysiologic mechanism that maintains atrial fibrillation by local conduction disturbance^4^. Serologic and imaging tests that are able to detect atrial fibrosis have been found to be correlated with recurrence of atrial fibrillation^5,6^. Recently, serum galectin-3 has been refocused to reveal cardiac fibrosis and predict rhythm outcomes after ablation^7^. We assessed the clinical implications of serum galectin-3 in patients who underwent catheter ablation for atrial fibrillation.

## Methods

### Study design and participants

This was a prospective non-randomized study. Consecutive patients who were planning to undergo a catheter ablation for atrial fibrillation were enrolled from February 2017 to September 2017 in a tertiary referral center. The Ethics Committee of the Korea University Anam Hospital Institutional Review Board approved this study. The protocol was consistent with the ethical guidelines of the 2008 Declaration of Helsinki. Written informed consent was obtained from all participants before enrollment. Because galectin-3 secretion in fibroblasts and macrophages was increased in diseases associated with fibrosis other than cardiac diseases, patients who were more than 80 years of age or had a history of liver cirrhosis, renal impairment (< 30 mL/min calculated by the Cockcroft-Gault equation), lung disease, or cancer were excluded to reduce confounding factors. Patients who had a history of prior catheter ablation for atrial fibrillation were also excluded to evaluate the relationship between spontaneous cardiac fibrosis and serum galectin-3 level. The study was registered in CRIS (KCT0005496).

### Cardiac imaging and serum galectin-3

Patients were examined on a 3-T magnetic resonance scanner (Achieva; Philips Medical Systems, Best, Netherlands) using a 32-element phased-array coil. Late gadolinium enhancement imaging was acquired using a 3-D inversion recovery-prepared, respiration-navigated, electrocardiography-gated, gradient-echo pulse sequence after the Look-Locker sequence to identify the optimal nulling time for the normal left ventricular myocardium. Pixels that showed increased enhancement in the left atrial wall were identified using the full width at half of the maximum technique and at 6-, 5-, 4-, 3-, and 2-standard deviations above the mean signal value of the unenhanced LV wall^8^. The volume of late gadolinium enhancement in the left atrium was defined as the summation of enhanced pixels over all slices.

Although there is no consensus on the preferred site for obtaining blood for measuring the level of serum galectin-3, we collected blood samples from the left atrium pertaining to the study protocol^7,9,10^. Serum levels of galectin-3 were measured using enzyme-linked immunosorbent assay kits (Galectin-3 Quantikine; R & D Systems, Inc., MN, U.S.). The mean minimum detectable dose was 0.016 ng/mL, which was determined by the intensity of color.

### 3-D electroanatomical voltage mapping and catheter ablation

An internal cardioversion was performed when the initial rhythm was atrial fibrillation to obtain information about the endocardial voltage during sinus rhythm. Using intracardiac multielectrode catheters positioned in the right atrium and the distal coronary sinus, 3–10 Jules of energy was delivered from an external defibrillator via junction box that allowed multielectrode catheter to be switched between routine diagnostic electrophysiology study and cardioversion. The 3-D mapping system (EnSite NavX System, Abbott, IL; CARTO system, Biosense Webster Inc., Diamond Bar, CA, U.S.) was used with a circular multielectrode catheter (AFocus II, St. Jude Medical, Inc., St. Paul, MN; Lasso, Biosense Webster, Inc., Diamond Bar, CA, USA) which generated an electroanatomical geometry with endocardial voltage information. The voltage ranged from 0.5 to 3.0 mV and was color-coded from grey to purple. The low voltage area was defined to be areas less than 0.5 mV in accordance with previous studies^11–14^. The mean atrial cycle length during atrial fibrillation was calculated using intervals of consecutive 20 signals acquired from each pulmonary vein.

Radiofrequency catheter ablation was performed using an open irrigated tip catheter (CoolFlex, St. Jude Medical, Minnetonka, MN; ThermoCool SmartTouch, Biosense Webster Inc., Diamond Bar, CA, U.S.). In patients with paroxysmal atrial fibrillation, the presence of a reproducible trigger that initiated atrial fibrillation was identified using high-frequency burst pacing and high-dose isoproterenol infusion before ablation. All patients had electrical isolation of the pulmonary veins and elimination of non-pulmonary vein triggers. Patients with non-paroxysmal atrial fibrillation had substrate modification focused on the automated complex fractionated atrial electrogram, or linear lesion, after pulmonary vein isolation, until atrial tachyarrhythmia could not be induced by burst pacing.

### Follow-up

Ambulatory monitoring and 12-lead electrocardiography were performed for 1–2 days after procedure during the patient’s hospital stay. An electrocardiography was routinely performed at each visit in the outpatient clinic, and 24-/48-Holter was performed at 1, 3, 6, 9, and 12 months, and then every 6 months thereafter. The physician determined whether antiarrhythmic drugs should be discontinued after 90 days (blanking period). Recurrence was defined as the occurrence of any sustained atrial tachyarrhythmia after a blanking period.

### Statistical analysis

Continuous variables are presented as the mean and standard deviation and were analyzed using the Student’s t-test or the non-parametric Wilcoxon rank sum test. Categorical variables are presented as the number and percentage and were analyzed using a χ^2^ test or Fisher’s exact test. Pearson’s correlation coefficients demonstrated the strength of the relationship between variables. Multivariable Cox regression analysis was used to establish the predictive factors for recurrence. The adjusted factors were age, sex, body mass index, comorbidities (i.e. congestive heart failure, hypertension, diabetes mellitus), clinical patterns of atrial fibrillation, hematocrit, renal function, left atrial diameter, late gadolinium enhancement in cardiac magnetic resonance imaging, and serum galectin-3 levels. The proportional hazard assumption was evaluated using log minus log survival curves, which were approximately parallel during the follow-up period. Statistical analyses were performed using SPSS (version 25, SPSS Institute, Inc., Chicago, IL, USA). All tests of significance were two-tailed. A *p* value of < 0.05 indicated statistical significance.

## Results

### Baseline characteristics

**Figure 1:**
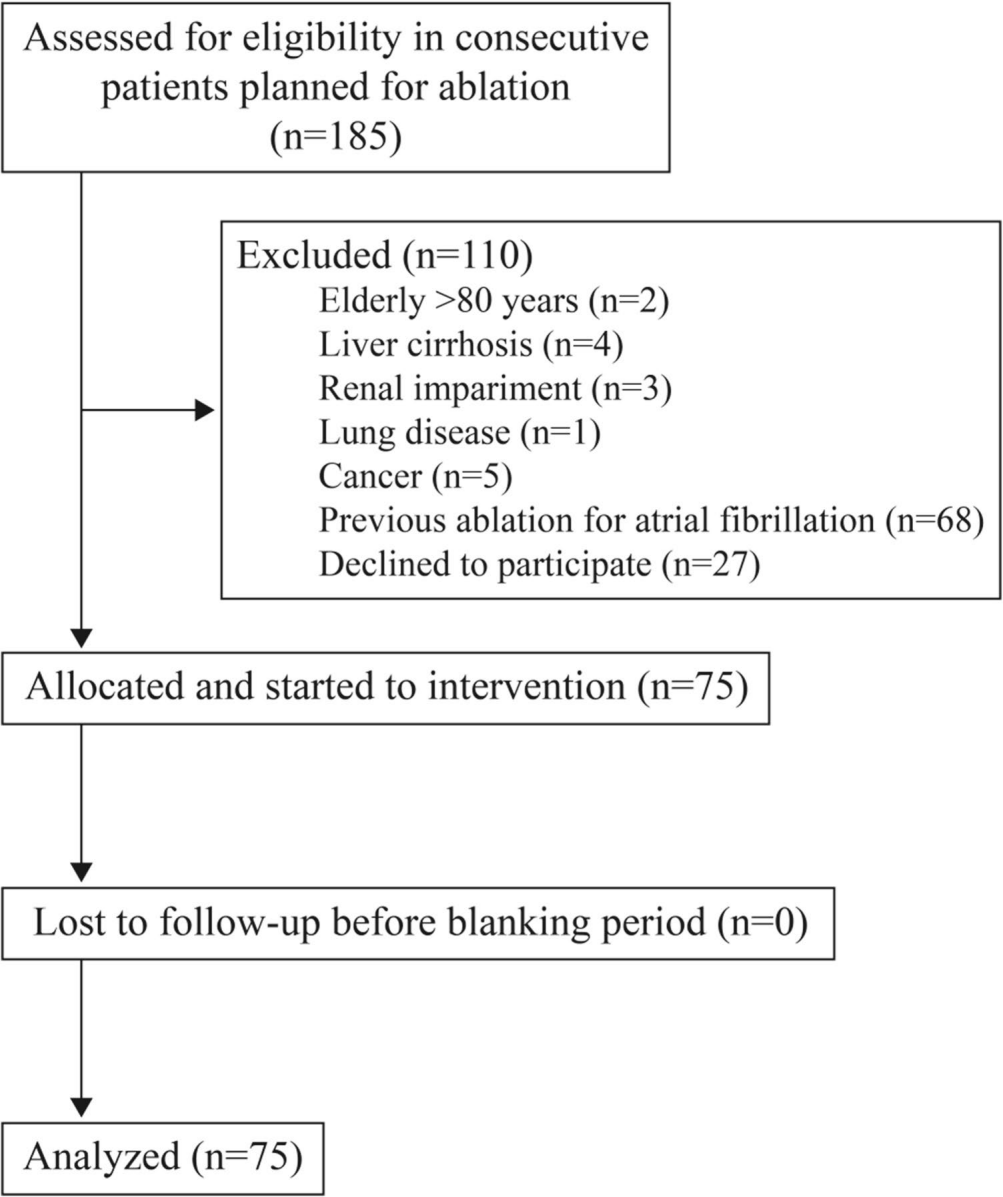
Flowchart.

Among 185 consecutive patients that were observed during the enrollment period, 75 patients were included in this study (Fig. 1). The mean age was 58 years, the proportion of females was 21% and the proportion with non-paroxysmal atrial fibrillation was 47%. The mean CHA_2_DS_2_-VASc score was 1.8, and the mean left atrial diameter was 41 mm on transthoracic echocardiography (Table 1).

**Table 1:** Baseline characteristics for recurrence after catheter ablation.

	Total (n = 75)	No recurrence (n = 55)	Recurrence (n = 20)	*p*
Age, years	57.8 ± 10.7	57.5 ± 10.6	58.6 ± 11.4	0.708
Female	16 (21.3)	14 (25.5)	2 (10.0)	0.208
Body mass index, kg/m^2^	25.3 ± 2.9	25.3 ± 2.6	25.5 ± 3.5	0.765
Non-paroxysmal atrial fibrillation	35 (46.7)	21 (38.2)	14 (70.0)	0.019
Congestive heart failure	31 (41.3)	24 (43.6)	7 (35.0)	0.600
Hypertension	34 (45.3)	27 (49.1)	7 (35.0)	0.307
Diabetes mellitus	7 (9.3)	3 (5.5)	4 (20.0)	0.077
Prior stroke	11 (14.7)	8 (14.5)	3 (15.0)	1.000
Creatinine clearance, mL/min	83.8 ± 22.9	84.5 ± 23.9	81.9 ± 20.5	0.665
CHA_2_DS_2_-VASc score	1.8 ± 1.3	1.8 ± 1.4	1.7 ± 1.3	0.600
Serum galectin-3, ng/mL	7.3 ± 2.1	6.9 ± 2.0	8.4 ± 2.1	0.004
Left atrial diameter, mm	40.8 ± 5.9	40.1 ± 5.1	42.9 ± 7.6	0.078
Late gadolinium enhancement, % (of left atrium)	15.9 ± 7.5	15.1 ± 7.0	17.9 ± 8.6	0.165
QRS complex duration, ms	90.9 ± 14.3	92.2 ± 14.3	87.3 ± 14.1	0.196
Mean cycle length of atrial fibrillation at pulmonary veins, ms	167.7 ± 47.2	177.2 ± 54.2	149.8 ± 23.4	0.165
Low voltage area (< 0.5 mV), % (of left atrium)	10.8 ± 14.8	8.4 ± 7.4	20.2 ± 28.9	0.231

Data are presented as n (%) or mean ± standard deviation.

### Serum galectin-3 and cardiac remodeling

**Figure 2:**
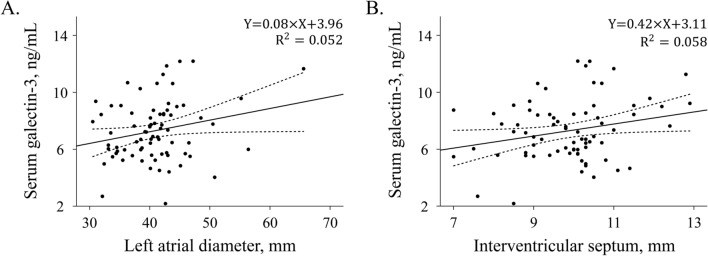
Scatter plot with Pearson correlation analysis. Significant positive correlation was found between galectin-3 and left atrial diameter (**A**), serum galectin-3 and left interventricular septum (**B**).

Serum galectin-3 obtained from the left atrium was positively correlated with left atrial size measured by anteroposterior diameter in the long-axis parasternal view on echocardiography (r = 0.23, *p* = 0.049, Fig. 2A), the volume in magnetic resonance imaging (r = 0.48, *p* < 0.001), and total low voltage area in a 3-D mapping system (r = 0.33, *p* = 0.021). Serum galectin-3 was positively correlated with left ventricular thickness on echocardiography (r = 0.24, *p* = 0.038, Fig. 2B). The low voltage area relative to the total left atrial wall (r = 0.25, *p* = 0.075), the proportion of area with late gadolinium enhancement to total left atrial area (r = 0.20, *p* = 0.079) and hematocrit (r = 0.22, *p* = 0.061) did not demonstrate significant relationships with galectin-3. There was no significant relationship between galectin-3 and the atrial cycle length during atrial fibrillation (*p* = 0.360). Among paired groups of patients who were sorted according to the type of atrial fibrillation or comorbidities, serum galectin-3 was higher in the diabetic group than in the non-diabetic group (9.52 ± 2.36 versus 7.07 ± 1.98, *p* = 0.003) but was not different in other paired groups.

### Recurrence after catheter ablation

**Figure 3:**
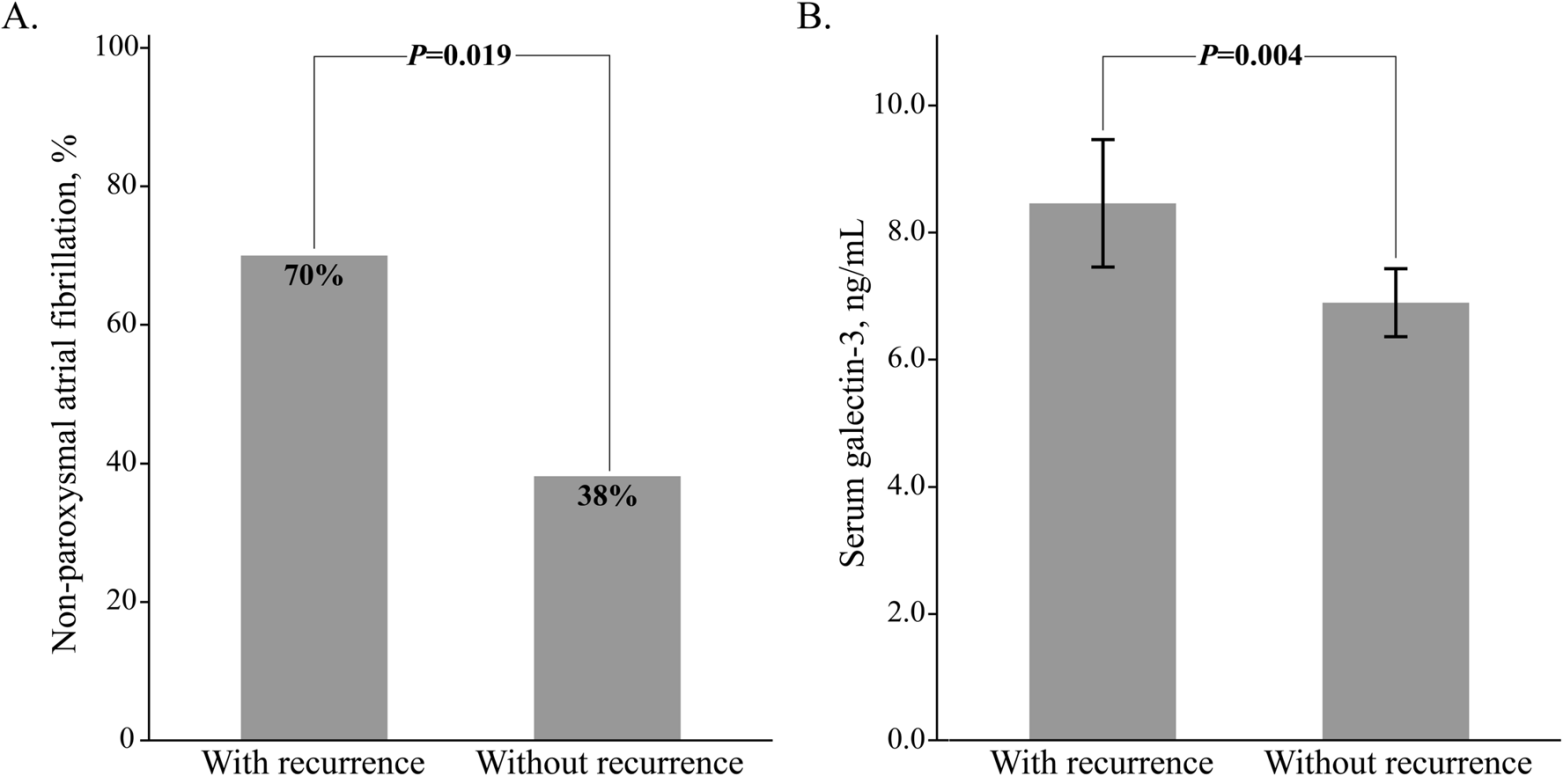
Bar graph comparing clinical factors, based on recurrence. Patients with recurrence showed higher proportions of non-paroxysmal atrial fibrillation (**A**) and higher serum galectin-3 levels (**B**) than patients without recurrence. Values are proportions or means with standard deviations.

Over the median follow-up period of 13.5 months, 20 recurrences (27% of cases) of atrial tachyarrhythmias (15 atrial fibrillation and 5 atrial tachycardia) were identified. After adjusting for multiple clinical factors, non-paroxysmal atrial fibrillation (hazard ratio 3.37; 95% confidence interval 1.29–8.83; *p* value 0.013) and serum galectin-3 level (hazard ratio 1.39; 95% confidence interval 1.14–1.70; *p* value 0.001) were found to be associated with an increased risk of recurrence (Table 2). Assessment of baseline characteristics indicated that patients with recurrence had higher proportions of non-paroxysmal atrial fibrillation and higher levels of serum galectin-3 compared to patients without recurrence (Fig. 3).

**Table 2:** Univariate and multivariable Cox regression analysis for recurrence after catheter ablation.

	Unadjusted HR (95% CI)	*p*	Adjusted HR (95% CI)*	*p*
Age, year	1.01 (0.97–1.06)	0.561		
Female	0.42 (0.10–1.82)	0.247		
Body mass index, kg/m^2^	1.02 (0.86–1.20)	0.837		
Congestive heart failure	0.68 (0.27–1.71)	0.411		
Hypertension	0.62 (0.25–1.56)	0.311		
Diabetes mellitus	3.21 (1.07–9.62)	0.037		
Non-paroxysmal atrial fibrillation	3.04 (1.17–7.91)	0.023	3.37 (1.29–8.83)	0.013
Hematocrit, %	1.02 (0.92–1.13)	0.774		
Creatinine clearance, mL/min	0.99 (0.97–1.02)	0.584		
Serum galectin-3, ng/mL	1.38 (1.12–1.70)	0.002	1.39 (1.14–1.70)	0.001
Left atrial diameter ≥ 43 mm	2.59 (1.07–6.26)	0.035		
Late gadolinium enhancement, %	1.05 (0.99–1.12)	0.115		

*Adjusted for all covariates listed in the table using the Cox proportional hazards regression model with backward stepwise Wald elimination.

*CI* confidence interval, *HR* hazard ratio.

## Discussion

This was a prospective cohort study to evaluate the prognostic clinical factors for recurrence after catheter ablation in patients with atrial fibrillation. Among potential risk factors, non-paroxysmal patterns of atrial fibrillation and high serum galectin-3 levels were predictive for increased recurrence of atrial tachyarrhythmias. Serum galectin-3 was related to left atrial enlargement and left ventricular hypertrophy, suggesting a relationship with cardiac structural remodeling. In addition, serum galectin-3 was higher in diabetic patients.

Over the last two decades, after the identification of pulmonary vein triggers, various ablation strategies have been suggested to improve ablation outcomes. However, two common strategies that target arrhythmogenic substrate, in addition to pulmonary vein isolation, were not superior to pulmonary vein isolation alone in patients with non-paroxysmal atrial fibrillation^15^. Therefore, selection of optimal candidates that are likely to benefit from ablation with limited efficacy is important for an individualized approach.

Left atrial enlargement has been reported to be a powerful predictor for recurrence after catheter ablation^16^. Pressure and volume overloads that are caused by congestive heart failure, coronary arterial disease, aging, obstructive sleep apnea, obesity, structural heart disease, and inflammation can lead to atrial remodeling^17^. In the present study, an enlarged left atrium (≥ 43 mm) was a statistically significant predictor for recurrence in univariate analysis. However, additional analysis with the stepwise variable selection of the multivariable Cox proportional hazards model did not confirm this finding. However, serum galectin-3 and non-paroxysmal atrial fibrillation were predictors in the final model. Because serum galectin-3 levels were positively correlated with left atrial size, more powerful predictors between galectin-3 and left atrial size might be included in the final model.

Fibrotic transformation of cardiomyocytes and interstitial accumulation of fibrotic tissue contribute to the development of arrhythmogenic substrates by slowing electrical conduction and enhancing anisotropy. Therefore, quantitative evaluation of the extent of atrial fibrosis may be helpful for predict rhythm outcomes after ablation. Instead of an endomyocardial biopsy which can have procedure-related complications and a low diagnostic yield, alternatives for assessing the extent of atrial fibrosis have been investigated in patients with atrial fibrillation. Early studies using magnetic resonance imaging reported that the late gadolinium enhanced area was positively correlated with myocardial collagen in the infarcted ventricle^18,19^. Expanding this approach to the atrium, Marrouche et al.^6^ reported the value of atrial late gadolinium enhancement for predicting recurrence after ablation. However, compared to ventricular imaging, atrial imaging has limited to assess myocardial fibrosis because the spatial resolution of the left atrial wall is insufficient, and is 2–3 times thinner than the left ventricular wall. Low voltage area (< 0.5 mV) that reflects conduction abnormalities caused by fibrosis has also been considered as a therapeutic approach^20^. However, the bipolar electrogram amplitude can be influenced by multiple factors that include the activation vector, angle of incidence, recording electrode size, interelectrode spacing, tissue contact, signal filtering, mapping density, and mapping resolution^21^.

Galecin-3 is a member of the animal lectins family, which is secreted by macrophages^22^. Because galectin-3 is implicated in inflammation and fibrosis in multiple organs, including the heart, liver, kidneys, lungs, spleen, ovary, and vessels^10^, we excluded patients with extracardiac dysfunction to reduce confounding bias. Interestingly, higher level of serum galectin-3 was seen in patients with thicker interventricular septum. This is not far from the result of previous studies which showed positive correlation between serum galectin-3 and diastolic dysfunction or ventricular mass index^9,23^. We presume that diastolic dysfunction may affect the serum galectin-3 level more than the degree of cardiac galectin-3 deposits does in patients with atrial fibrillation. Also, serum galectin-3 level had a weak correlation with either left atrial diameter or interventricular septal thickness in our study. Therefore, we could not discern the origin of serum galectin-3 between the atrium and ventricle. However, because atrial fibrillation is a progressive disease with structural remodeling of both the atrium and the ventricle, this would not change the clinical implication of serum galectin-3. Furthermore, serum galectin-3 has been associated with myocardial fibrosis and increased mortality in patients with heart failure^24^. Recently, inhibition of galectin-3 reduced atrial fibrosis and the total burden of atrial fibrillation^7^.

Compared with peripheral blood sampling, the invasive intracardiac sampling required by our model limits its utility for pre-procedural assessment. However, galectin-3 levels in peripheral blood were higher and less predictive of recurrence after ablation compared with those in intracardiac blood^25^. Galectin-3 from peripheral blood could be affected by other pathologies, such as cancer or non-cardiac fibrotic diseases^25^. Therefore, a further study to evaluate the clinical value of galecin-3 from peripheral blood is needed.

## Limitations

There were several limitations to this study. First, this was a single center study with a small number of patients and a short duration of follow-up. However, all study patients were consecutively enrolled during the study period. Second, histopathological data for cardiac tissue was not available and could not be evaluated.

## Conclusion

In summary, elevated serum galectin-3 was related to the left atrial enlargement and increased risk of recurrence after radiofrequency catheter ablation in patients with atrial fibrillation. Therefore, serum galectin-3 could be used as a biomarker for evaluation of the atrial remodeling severity and post-ablative prediction of recurrence in patients with atrial fibrillation.

## Data Availability

The data that support this article will be shared on reasonable request with the corresponding author.
